# Post-Basal Meningitis Due to Pneumococcus Lanceolatus

**Published:** 1904-03-26

**Authors:** 


### POST-BASAL MENINGITIS DUE TO PNEUMOCOCCUS LANCEOLATUS.

Dr. J. P. Parkinson [1] reports the case of a child aged two years who was admitted to hospital with a temperature of 104·8° F., pulse 141, and respirations 70. Signs of pneumonic consolidation at the right apex were present. There was a history of four days' illness with fever, vomiting and cough. The physical signs in the right lung persisted for about five weeks, when they gradually resolved. A few days after the child's admission, rigidity of the muscles at the back of the neck was noticed. This symptom increased, the occiput becoming markedly retracted; and gradually complete opisthotonos of the trunk muscles supervened, the legs being rigid and extended, the arms rigid, flexed at the elbows, with forearms pronated and fingers flexed over the adducted thumb. The patellar reflexes could just be obtained. Kernig's sign was not well marked. The fontanelle was slightly bulged, and the fundus of the eye showed no abnormal changes, though the child appeared to be blind as visual reflexes were absent. A fortnight after admission half an ounce of clear fluid was withdrawn from the spine by lumbar puncture by Mr. Rhodes, senior resident medical officer. It issued under increased pressure, and was clear, forming a flocculent cloud later on. It contained a trace of albumen. Examination of the fluid showed the presence of *Diplococcus lanceolatus* in pure culture. The condition of the child remained unchanged for about five weeks, with diarrhœa and occasional vomiting. The lung signs then gradually cleared up, and a month later the opisthotonos diminished and the sight returned. Soon the child began to sit up in bed and to take an interest in his surroundings, and he developed that voracious appetite described by Mr. Stephen Paget as sometimes occurring after cerebral injury or disease. Ultimately he appeared to have completely recovered, showing no traces of disease, either physical or mental.

In commenting on the case Dr. Parkinson remarks that the chief interest lies in the fact that the cerebral symptoms, though clinically those of a typical case of posterior basic meningitis, appear to have been due to the *Diplococcus lanceolatus* and not to the *Diplococcus intracellularis.* In most cases of pneumococcal meningitis complicating pneumonia the vertex of the brain is affected and a fatal result is said to be invariable. This case shows that the inflammation may be chronic and situated at the posterior part of the base, and that recovery may be possible. It also shows the value of lumbar puncture when cerebral symptoms appear during the course of pneumonia and other infective diseases.

[1] Brit. Jour. Child. Dis. March 1, 1904.

